# 

**Published:** 1888-07

**Authors:** 


### TABLE OF CONTENTS

### Original and Selected Articles.

Notes in the practice of G. G. Roy, M.D., Atlanta, Georgia ..... 241
Spoon-handle obstetric forceps, by J. Stainback Wilson, M.D., Atlanta, Ga ..... 245
Recent progress in obstetrics and gynecology in Great Britain, by E. S. McKee, M.D., Cincinnati, Ohio ..... 247
Electrolysis in stricture—report of three cases with remarks, by J. J. Berry, M.D., Portsmoth, N H ..... 250
Relation of the use of coffee to susceptibility to typhoid fever, by W. A. Cusick. M.D., Or... 252
Ogeechee Medical Association—stated bymonthly meeting, reported by R. Y. Lane, Secretary, March 8, 1888 ..... 253
Notes on the use of antiseptics in surgery with special reference to the pine product called sanitas, Frederick Bowerman Jesset, F.R.C.S. 255
Iodide of ammonium in asthma; a cure for wrinkles ..... 256

### Abstracts and Gleanings.

Tracheotomy in morphine poisoning ..... 257
Local application of calomel in phagedena ..... 258
Intubation of the larynx ..... 259
Intestinal obstruction relieved by laparotomy 260
Sulphur in chlorosis; salicylate of mercury in syphilis ..... 261
Antifebrin ..... 262
Influence of medicines taken by nursing women upon their infants; the treatment of mastitis ..... 263
Bacilli of intermittent fever ..... 264
Uterine hemorrhage in pregnancy; diphtheria carried by turkeys ..... 265
Creoline; jaborandi in obstetric practice ..... 266
The treatment of tonsilitis by salicylate of sodium; haya poison; confirmation of its anæsthetic powers ..... 267
Inter-cranial neuralgias or migraine; cocaine in the vomiting of pregnancy ..... 268
Communicability of disease from animals to man; oil of mullein in enuresis ..... 269
Reflex neuroses due to constipation; wart-charming extraordinary; acute rheumatism; a new elastic mucilage ..... 270

### Scientific Items.

The growth of science; magazine guns ..... 271
The great Yellowstone geyser now active; the Edison photophone; the sun as an incendiary ..... 272

### Practical Notes and Formulæ.

For piles and pruritus ani; misturia acidi muriatici; misturia anti-emetica; misturia rhei et calcis; compound mixture of muriate of ammonia; pile ointment; antipyrin in gonorrhœa ..... 273
Sore nipples; chronic bronchitis; hair tonic; hemorrhoids; hydrochloric acid and cocaine 274

### Editorials and Miscellaneous.

Our collaborators; dysentery ..... 275
The district committees; the American Rhinological Association ..... 276
Murdock's liquid food; chlor-anodyne; books and pamphlets received ..... 277
To medical students ..... 279
Special notes ..... 280

